# Efficacy and safety of apixaban versus warfarin in new-onset atrial fibrillation post coronary artery bypass grafting: A retrospective cohort study

**DOI:** 10.1097/MD.0000000000049695

**Published:** 2026-07-10

**Authors:** Rawan A. Bukhari, Majed Almutairi, Sultan Alraddadi, Omar S. Alkhezi, Mansour Alomran, Bijesh Kumar, Marwan A. Alrasheed, Hind Almodaimegh

**Affiliations:** a Pharmaceutical Care Services, King Salman Specialized Hospital, Taif, Saudi Arabia; b Pharmaceutical Care Services, King Abdulaziz Medical City, Riyadh, Saudi Arabia; c King Abdullah International Medical Research Center, Research Office, Riyadh, Saudi Arabia; d College of Pharmacy, King Saud Bin Abdulaziz University for Health Sciences, Riyadh, Saudi Arabia; e Department of Pharmacy Practice, College of Pharmacy, Qassim University, Qassim, Saudi Arabia; f Department of Cardiac Surgery, King Abdulaziz Medical City, Riyadh, Saudi Arabia; g College of Medicine, King Saud Bin Abdulaziz University for Health Sciences, Riyadh, Saudi Arabia; h Department of Biostatistics, King Abdullah International Medical Research Center, Riyadh, Saudi Arabia; i Department of Clinical Pharmacy, College of Pharmacy, King Saud University, Riyadh, Saudi Arabia.

**Keywords:** anticoagulant agent, anticoagulation, cardiac surgery, coronary artery bypass grafting, DOACs, postoperative atrial fibrillation, warfarin

## Abstract

New-onset postoperative atrial fibrillation (POAF) is a common complication following cardiac surgery, especially coronary artery bypass grafting (CABG), affecting up to 40% of patients, and is associated with increased risks of thromboembolic events and prolonged hospital stays. Traditionally, warfarin has been the standard anticoagulant for stroke prevention in atrial fibrillation. However, it requires regular international normalized ratio monitoring. Direct comparisons between apixaban and warfarin in the CABG population remain scarce. This study aimed to compare the effectiveness and safety of apixaban versus warfarin in patients with POAF after CABG. A retrospective observational cohort study was conducted at King Abdulaziz Medical City, Riyadh, from January 1, 2017, to December 31, 2023. Eligible patients aged 18 and older who developed new-onset POAF and received apixaban or warfarin within 7 days postsurgery were included. The primary outcome was the incidence of thromboembolic events, while the secondary outcomes were safety outcomes, including bleeding complications. Out of 1277 records, 143 patients met the inclusion criteria. The incidence of thromboembolic events within 90 days was slightly lower in the apixaban group (2.0%) compared to warfarin (7.0%), though not statistically significant (*P* = .137). Major bleeding events occurred in 6.0% of patients on apixaban and 7.0% on warfarin (*P* = .825), and minor bleeding rates were also not statistically different, with 7.0% for apixaban and 11.6% for warfarin (*P* = .360), indicating no difference in safety profiles. Apixaban and warfarin demonstrated similar efficacy in preventing thromboembolic events and comparable safety regarding bleeding complications in patients with POAF following CABG, supporting apixaban as a reasonable alternative in this population.

## 1. Introduction

Coronary artery bypass grafting (CABG) is a common and life-saving surgical intervention for patients with coronary artery disease.^[1]^ While CABG improves cardiac perfusion and reduces the burden of ischemic heart disease, it is not without complications. One such complication, atrial fibrillation (AF), frequently emerges in the postoperative period, representing a significant clinical challenge.^[2]^

New-onset postoperative AF (POAF) refers to the development of AF after CABG surgery. It is a common arrhythmia characterized by irregular and rapid atrial electrical activity and has been reported in up to 40% of patients undergoing CABG.^[3]^ The development of AF in this specific postoperative setting is multifactorial, involving factors such as inflammation, autonomic dysregulation, and the hemodynamic consequences of cardiac surgery.^[4]^ Beyond its immediate impact on cardiac function, POAF is associated with adverse outcomes. The irregular rhythm poses an increased risk of thromboembolic events, most notably stroke, due to stasis of blood in the fibrillating atria.^[4,5]^ POAF also complicates the postoperative course by contributing to hemodynamic instability, prolonged hospital stays, and increased healthcare costs.^[5]^

Anticoagulation strategies in POAF are critical for mitigating the associated risks. Traditionally, warfarin has been the standard anticoagulant medication used for stroke prevention in patients with AF.^[6]^ However, warfarin has several limitations, including a narrow therapeutic window, the need for regular monitoring of blood clotting levels measured by the international normalized ratio, and interactions with various foods and medications.^[7]^ Apixaban, a direct oral anticoagulant (DOAC), acts by selectively inhibiting factor Xa. Compared to warfarin, apixaban offers advantages in efficacy, safety, convenience, and fewer interactions.^[8]^

Despite the prevalence and clinical significance of POAF, there is limited research specifically addressing optimal anticoagulation management in this context. Recent studies have examined the efficacy and safety of DOACs in the post-cardiac surgery period.^[9,10]^ However, most of these studies have addressed general aspects of DOAC use after cardiac surgery, and direct comparisons between apixaban and warfarin remain limited. Manuel et al (2020) compared warfarin and DOACs in post-CABG AF, finding longer hospital stays and delayed therapeutic anticoagulation with warfarin.^[11]^ Safety outcomes varied, with 1 study reporting more interventions for pericardial effusions in the DOAC group and another indicating higher major bleeding in the warfarin group.^[11]^ A 2022 meta-analysis by Koh et al favored DOACs, reporting lower risks of neurological events and bleeding.^[9]^ Naik et al (2022) found similar safety between apixaban/rivaroxaban and warfarin; however, they emphasized the need for larger studies.^[12]^ Pereira et al (2023) demonstrated DOACs, especially rivaroxaban, as more cost-effective than warfarin in POAF with a 52% cost decrease, suggesting the need for larger trials to confirm their safety and efficacy.^[13]^ Therefore, we conducted this study comparing apixaban versus warfarin in patients with new-onset AF following CABG. The objectives were to evaluate the effectiveness of these anticoagulants in preventing thromboembolic events and assess the safety profiles, particularly bleeding complications.

## 2. Materials and methods

### 2.1. Place of study

This study was conducted at King Abdulaziz Medical City-Central Region, a tertiary healthcare hospital in Riyadh, Saudi Arabia. The proposal was approved by the Institutional Review Board at King Abdullah International Medical Research Center (IRB/0323/24). Demographic and clinical data for this study were sourced from the hospital information system and collected on a Microsoft Office Excel 2010 sheet protected by a password key to ensure confidentiality.

### 2.2. Study design and duration

**Figure 1.**
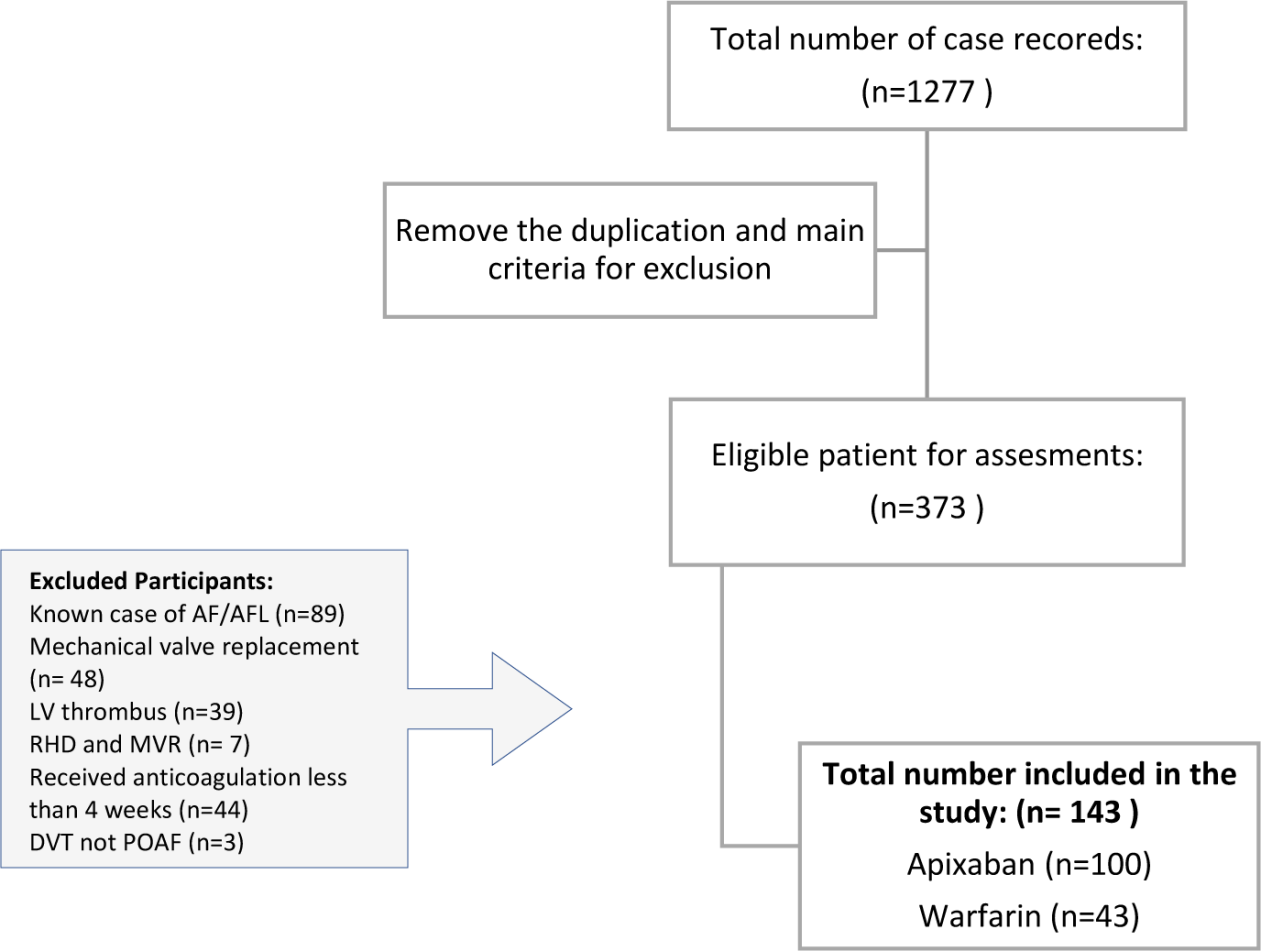
Patient selection flowchart showing inclusion and exclusion of study participants. AF = atrial fibrillation, AFL = atrial flutter, DVT = deep vein thrombosis, LV = left ventricle, MVR = mitral valve replacement, n = number of participants, POAF = postoperative atrial fibrillation, RHD = rheumatic heart disease.

This was a single-center, retrospective observational cohort study including all eligible patients treated between January 1, 2017, and December 31, 2023. The patient selection process is summarized in Figure 1, and baseline characteristics are presented in the “Patient demographics and clinical characteristics” section (Table 2).

### 2.3. Study definitions

Table 1.^[2,6,14,15]^

**Table 1:** Study definitions.

Definitions	
AF	A supraventricular tachyarrhythmia with uncoordinated atrial activation and ineffective atrial contraction. Electrocardiographic characteristics include irregular R-R intervals (when atrioventricular conduction is present), absence of distinct *P* waves, and irregular atrial activity, also known as fibrillatory waves.^[6]^
New-onset POAF	A new-onset AF (i.e., in a patient without a prior history of the arrhythmia) occurring early enough after surgery that persistent or recurrent AF during the index hospitalization, identified by telemetry and confirmed by ECG, anticoagulation was recommended to be continued for a minimum of 4 to 6 weeks.^[2]^
Hemodynamically stable	Defined by the AHA, includes (SBP > 90 mm Hg, no altered mental status, no cardiac ischemia, or severely decompensated heart failure due to the underlying rhythm)^.[14]^
ISTH major bleeding	Defined as fatal bleeding, symptomatic bleeding in a critical area or organ, bleeding causing a fall in hemoglobin level of 2g/dl or more, and/or bleeding leading to transfusion of 2 or more units of whole blood or red cells.^[15]^
Thromboembolic Events	Defined as ischemic stroke or TIA, VTE, or intracardiac thrombus
Major bleeding events	Such as gastrointestinal bleeding, intracranial bleeding, or major bleeding as defined by the ISTH
Minor bleeding:	Hematuria, hematoma, nasal bleeding (epistaxis), pharyngeal bleeding
CHA_2_DS_2_-VASc Score	A medical tool used to guide physicians on blood-thinning treatment to prevent stroke in people with non-valvular atrial fibrillation AF. Mnemonic stands for: Congestive heart failure, Hypertension, Age 75 years or older, Diabetes mellitus, previous Stroke/TIA, Vascular disease, Age 65–74 years, Sex category.^[6]^
HAS-BLED scores	A medical tool used to calculate the 1-year risk of major bleeding for people on blood-thinning drugs for AF, Mnemonic stands for: hypertension, abnormal renal and liver function, stroke, bleeding, labile INR, elderly, drugs or alcohol.^[6]^

AF = atrial fibrillation, AHA = American Heart Association, CHA_2_DS_2_-VASc = (C)ongestive heart failure, (H)ypertension, (Age ≥75 years), (D)iabetes mellitus, (Stroke/TIA), (V)ascular disease, (Age 65–74 years), (Sex category), ECG = electrocardiogram, HAS-BLED = (H)ypertension, (A)bnormal renal/liver function, (S)troke, (B)leeding history, (L)abile INR, (E)lderly (age >65 years), (D)rugs/alcohol concomitantly, INR = international normalized ratio, ISTH = International Society on Thrombosis and Hemostasis, POAF = postoperative atrial fibrillation, SBP = systolic blood pressure, TIA = transient ischemic attack, VTE = venous thromboembolism.

### 2.4. Criteria of inclusion and exclusion

#### 2.4.1. Inclusion criteria

The patients who met the following criteria were included in this study:

Aged 18 years or older, underwent CABG ± valve repair with subsequently developed new-onset POAF, persistent or recurrent during the index hospitalization, hemodynamically stable, and administered anticoagulation (apixaban or warfarin) within 7 days of surgery.

#### 2.4.2. Exclusion criteria

Patients with a history of AF or Flutter, rheumatic mitral stenosis or mechanical heart valves, infected endocarditis, hepatic disease with aberrant coagulation, left ventricular thrombus, triple-positive antiphospholipid syndrome, pregnant women, patients who had a stroke within 7 days, and those with homozygous factor V Leiden were excluded.

### 2.5. Endpoints

#### 2.5.1. Primary endpoints

##### 2.5.1.1. Efficacy outcomes

- Incidence of thromboembolic events during hospitalization or 90 days following discharge, or until the first follow-up appointment, and after 90 days until data extraction.

#### 2.5.2. Secondary endpoints

##### 2.5.2.1. Safety outcomes

- Incidence of major bleeding events, incidence of minor bleeding, and readmission or death within 90 days of discharge for a bleeding or thromboembolic event.

### 2.6. Statistical analysis

Data were entered using Excel. Continuous variables were presented as mean ± standard deviation and compared using *t*-tests, while categorical variables were presented as numbers and percentages and compared using Chi-square or Fisher exact tests, as appropriate. Multivariable logistic regression was performed to identify independent predictors of rehospitalization and pericardial effusion within 90 days after discharge. Statistical significance was defined as *P* < .05. All analyses were performed using statistical analysis system 9.4.

## 3. Results

### 3.1. Participant selection

Initially, 1277 case records were collected. After removing duplicates and applying exclusion criteria, 373 patients were evaluated for eligibility according to the predefined criteria. Of these, 143 satisfied the inclusion criteria (Fig. 1).

#### 3.1.1. Patient demographics and clinical characteristics

Patient demographics and clinical characteristics are summarized in Table 2. The study population consisted predominantly of male participants, with 80% in the apixaban group and 74.4% in the warfarin group. The mean age was similar between the apixaban (64.31 ± 7.86 years) and warfarin (63.77 ± 10.17 years) groups. The mean body mass index was 28.8 ± 4.56 kg/m^2^ in the apixaban group and 26.5 ± 4.95 kg/m^2^ in the warfarin group. The mean serum creatinine levels were 104.7 ± 82.5 μmol/L and 127 ± 80 μmol/L, respectively, while the mean estimated glomerular filtration rate was 81 ± 31.8 mL/min/1.73m^2^ in the apixaban group and 68.42 ± 35.7 mL/min/1.73m^2^ in the warfarin group.

**Table 2:** Descriptive table of demographic variables and clinical characteristics.

Variables	Apixaban (N = 100)	Warfarin (N = 43)	*P* value
	n (%), Mean ± SD, or Median [Q1, Q3]		
Gender			
Male	80 (80.0)	32 (74.4)	.46
Female	20 (20.0)	11 (25.6)	
Age (yrs)	64.31 ± 7.86	63.77 ± 10.17	.73
BMI (kg/m^2^)	28.8 ± 4.56	26.5 ± 4.95	.011
SCr (μmol)	104.7 ± 82.5	127 ± 80	.130
eGFR (mL/min/1.73 m^2^)	81 ± 31.8	68.42 ± 35.7	.034
Hgb (μmol)	99 ± 10.5	95.6 ± 11.5	.081
INR	1.07 ± 0.09	1.05 ± 0.07	.252
PT	11.7 ± 1.3	11.6 ± 1.5	.607
APTT	31.3 ± 6.6	29.9 ± 4.4	.220
Hypertension	79 (79.0)	32 (74.4)	.547
Diabetes mellitus	83 (83.0)	29 (67.4)	.038
Dyslipidemia	58 (58.0)	15 (34.9)	.011
CKD	16 (16.0)	13 (30.2)	.052
History of Stroke	10 (10.0)	2 (4.7)	.290
Heart failure	10 (10.0)	13 (30.2)	.003
LVEF before surgery			
LVEF ≤ 40%	38 (38.0)	23 (53.5)	.042
LVEF 41–49%	19 (19.0)	11 (25.6)	
LVEF ≥ 50%	43 (43.0)	9 (20.9)	

APTT = activated partial thromboplastin time, BMI = body mass index, CKD = chronic kidney disease, eGFR = estimated glomerular filtration rate, Hgb = hemoglobin, INR = international normalized ratio, LVEF = left ventricular ejection fraction, PT = prothrombin time, SCr = serum creatinine, SD = standard ratio.

In terms of comorbidities, the apixaban group had a greater prevalence of dyslipidemia (58.0% vs 34.9%, *P* = .011). Heart failure was more common in the warfarin group than in the apixaban group (30.2% in warfarin vs 10.0% in apixaban, *P* = .003). Additionally, the apixaban group had a greater proportion of patients with diabetes mellitus (83.0% vs 67.4%, *P* = .038) compared to the warfarin group.

#### 3.1.2. Surgery and AF characteristics

The type of cardiac surgery differed significantly between the apixaban and warfarin groups (*P* < .001). The apixaban group had a greater proportion of patients who underwent isolated CABG procedures. In contrast, the warfarin group had more patients receiving CABG with mitral valve repair (37.2%) or CABG with mitral and tricuspid valve repair (11.6%) (Table 3).

**Table 3:** Postoperative variables according to AF following cardiac surgery.

Variables	Apixaban (N = 100)	Warfarin (N = 43)	*P* value
	n (%)	n (%)	
Type of coronary artery bypass grafting CABG surgery			
Isolated CABG for 2-vessel disease	10 (10.0)	3 (7.0)	< .001
Isolated CABG for 3-vessel disease	70 (70.0)	13 (30.2)	
Isolated CABG for 4-vessel disease	20 (20.0)	3 (7.0)	
CABG + Mitral valve repair	0 (0.0)	16 (37.2)	
CABG + Mitral valve repair+ + Tricuspid valve	0 (0.0)	5 (11.6)	
CABG + Tricuspid valve repair	0 (0.0)	3 (7.0)	
Type of AF			
Paroxysmal or Persistent > 48 h	84 (84.0)	32 (74.4)	.179
Recurrent	16 (16.0)	11 (25.6)	
Received presurgery			
Beta-blocker	84 (84.0)	32 (74.4)	.234
Non-dihydropyridine calcium blocker	0 (0.0)	1 (2.3)	
Amiodarone	0 (0.0)	0 (0.0)	
Pharmacological agents after AF			
Beta-blocker	13 (13.0)	8 (18.6)	.079
Amiodarone	11 (11.0)	10 (23.3)	
Amiodarone with Beta-blocker	76 (76.0)	25 (58.1)	
CHA_2_DS_2_VASc Score			
Low risk (0)	3 (3.0)	1 (2.3)	.701
Moderate risk (1–2)	37 (37.0)	13 (30.2)	
High risk (≥ 3)	60 (60.0)	29 (67.4)	
HAS-BLED scores			
Low risk (0–2)	88 (88.0)	22 (51.2)	< .001
High risk (≥ 3)	12 (12.0)	21 (48.8%)	

AF = atrial fibrillation, CABG = coronary artery bypass grafting, HAS-BLED = (H)ypertension, (A)bnormal renal/liver function, (S)troke, (B)leeding history, (L)abile INR, (E)lderly (age >65 years), (D)rugs/alcohol concomitantly, N/n = number of participants.

Regarding the type of AF, the groups were similar, with most patients having paroxysmal or persistent AF lasting over 48 hours (84.0% in apixaban vs 74.4% in warfarin, *P* = .179). The proportion of patients receiving beta-blocker therapy was also comparable between the apixaban (84.0%) and warfarin (74.4%) groups (*P* = .234).

The CHA_2_DS_2_-VASc – (C)ongestive heart failure, (H)ypertension, (Age ≥75 years), (D)iabetes mellitus, (Stroke/TIA), (V)ascular disease, (Age 65–74 years), (Sex category) – scores showed the majority of patients in both groups were at high risk (≥ 3), with 60.0% in the apixaban group and 67.4% in the warfarin group. However, the HAS-BLED – (H)ypertension, (A)bnormal renal/liver function, (S)troke, (B)leeding history, (L)abile INR, (E)lderly (age >65 years), (D)rugs/alcohol concomitantly – scores indicated a greater proportion of patients who were at low risk (0–2) in the apixaban group (88.0%) compared to the warfarin group (51.2%, *P* < .001).

#### 3.1.3. Postoperative medications

Table 4 presents the discharge medication profiles for patients in the apixaban and warfarin groups. Antiplatelet agents were frequently prescribed, with the majority of patients in both groups receiving aspirin (84.0% apixaban, 97.7% warfarin). Clopidogrel use was more frequent in the apixaban group (11.0% vs 2.3%). Regarding renin-angiotensin-aldosterone system medications, a greater proportion of warfarin patients were prescribed angiotensin-converting enzyme inhibitors (44.0% vs 30.0%), while angiotensin-receptor blockers were only prescribed in the apixaban group (7.0% vs 0%). Statins were frequently prescribed to both groups.

**Table 4:** Discharge medications.

Variables	Apixaban (N = 100)	Warfarin (N = 43)	*P* value
Discharge medications			
Antiplatelet			
Aspirin, n (%)	84 (84.0)	42 (97.7)	.140
Clopidogrel, n (%)	11 (11.0)	1 (2.3)	
Aspirin and clopidogrel, n (%)	3 (3.0)	0 (0.0)	
RAAS medications			
ACEIs, n (%)	30 (30.0)	19 (44.0)	
ARB, n (%)	7 (7.0)	0 (0.0)	
ARNI, n (%)	2 (2.0)	0 (0.0)	
Beta-blocker, n (%)	94 (94.0)	43 (100.0)	.101
Dihydropyridine Calcium Channel Blocker, n (%)	11 (11.0)	5 (11.6)	
Antiarrhythmic:			
Amiodarone, n (%)	29 (29.0)	8 (18.6)	.005
Digoxin, n (%)	4 (12.1)	0 (0.0)	
Dyslipidemia medications:			
Statin, n (%)	93 (98.9)	36 (83.7)	.042
Statin and Ezetimibe, n (%)	1 (1.1)	3 (7)	

ACEIs = angiotensin-converting enzyme inhibitors, ARB = angiotensin-receptor blocker, ARNI = angiotensin-receptor/neprilysin inhibitor, RAAS = renin-angiotensin-aldosterone system, N/n = number of participants.

### 3.2. Primary endpoint

**Figure 2.**
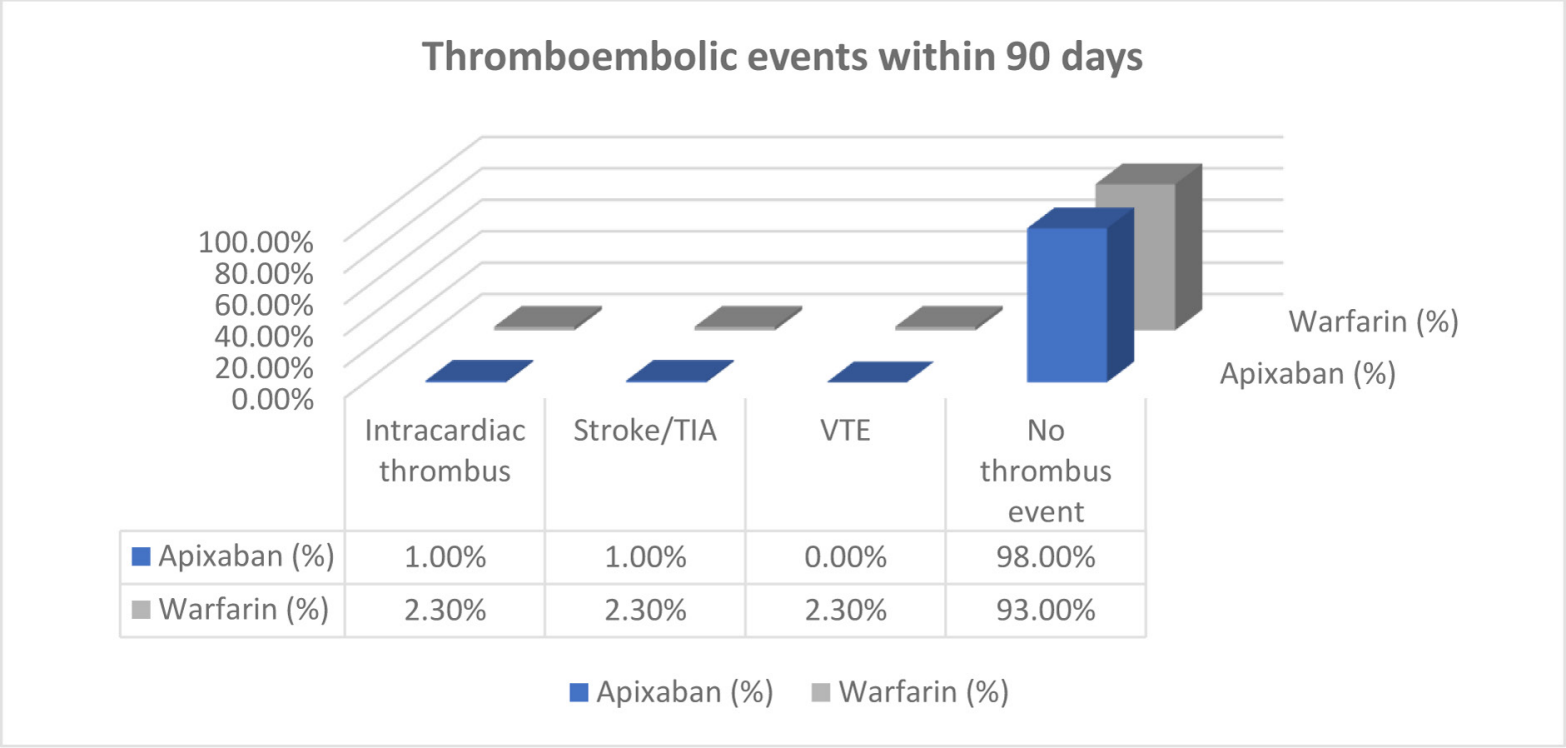
Thromboembolic events within 90 days following POAF and initiation of anticoagulation. POAF = postoperative atrial fibrillation, TIA = transient ischemic attack, VTE = venous thromboembolism.

The primary outcome of this study was the rate of thromboembolic events between patients treated with apixaban and those treated with warfarin within the first 90 days (Fig. 2). The incidence of thromboembolic events was slightly lower in the apixaban group (2.0%) compared to the warfarin group (7.0%), though this difference did not reach statistical significance (*P* = .137).

**Figure 3.**
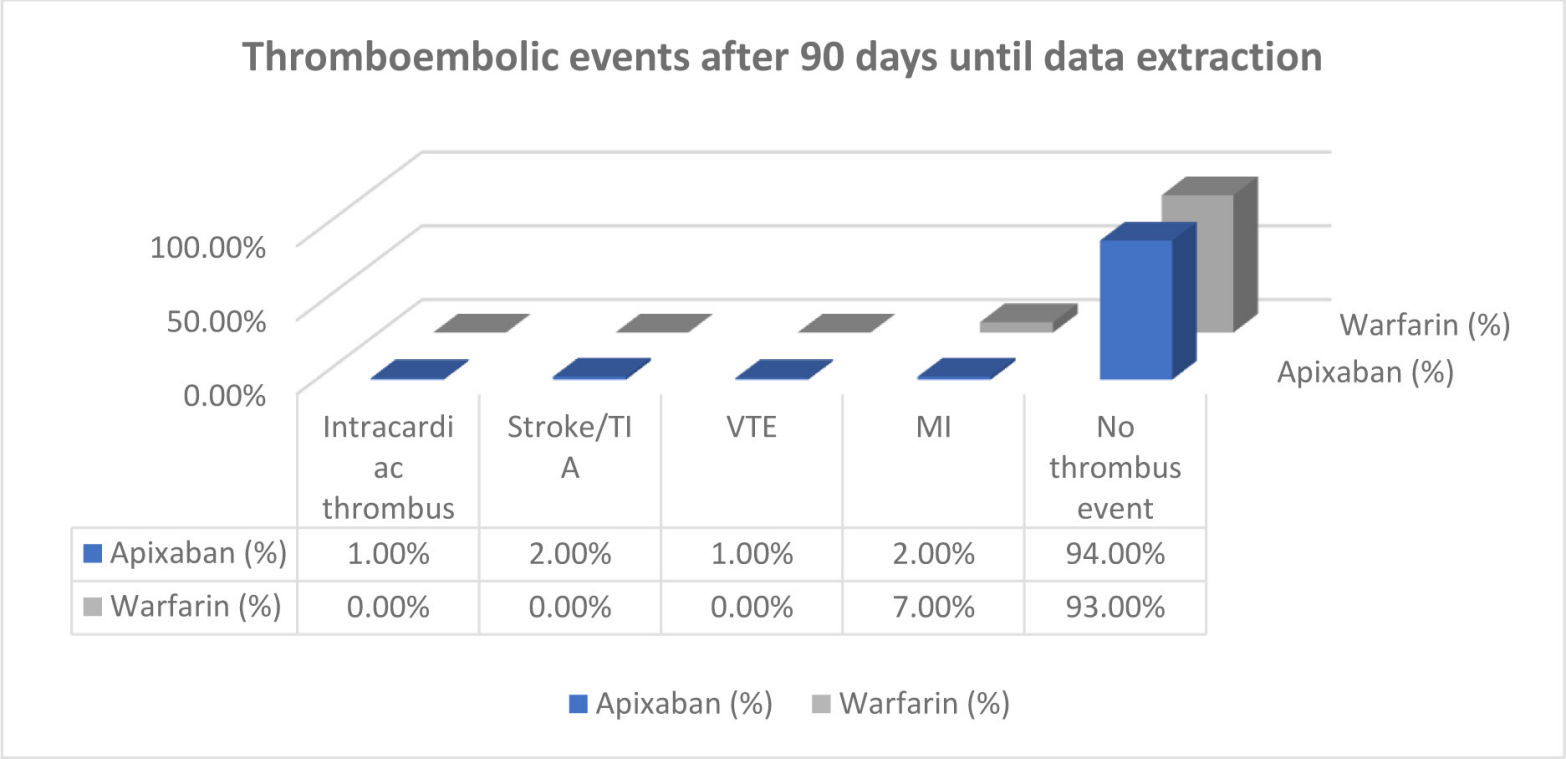
Thromboembolic events after 90 days following POAF until data extraction. MI = myocardial infarction, POAF = postoperative atrial fibrillation, TIA = transient ischemic attack, VTE = venous thromboembolism.

For thromboembolic events after 90 days until data extraction (Fig. 3), there was no difference in the rates between the 2 groups (6.0% for apixaban and 7.0% for warfarin); however, warfarin patients experienced more myocardial infarction events, while apixaban patients had more strokes/ transient ischemic attacks and venous thromboembolism events.

Overall, there was no significant difference between the groups for thromboembolic events at both time points (within 90 days and after 90 days), suggesting similar outcomes for both anticoagulants in preventing thromboembolic complications.

Comparing CHA_2_DS_2_-VASc Scores between apixaban and warfarin groups (Table 5) along with the occurrence of any thrombotic events across different CHA_2_DS_2_-VASc risk categories

**Table 5:** CHA_2_DS_2_-VASc scores comparison between apixaban and warfarin groups in thrombotic events.

Groups	CHA_2_DS_2_VASc Score			*P* value
	Low risk (0)	Moderate risk (1–2)	High risk (≥ 3)	
	n (%)	n (%)	n (%)	
Apixaban (N = 100), Total	3 (3.0)	37 (37.0)	60 (60.0)	
Any thrombotic event	0 (0.0)	2 (5.4)	0 (0.0)	.176
Warfarin (N = 43), Total	1 (2.3)	13 (30.2)	29 (67.5)	
Any thrombotic event	0 (0.0)	1 (7.7)	2 (6.9)	.958

CHA_2_DS_2_-VASc = (C)ongestive heart failure, (H)ypertension, (Age ≥75 years), (D)iabetes mellitus, (Stroke/TIA), (V)ascular disease, (Age 65–74 years), (Sex category), N/n = number of participants.

The distribution of CHA_2_DS_2_-VASc scores among patients receiving Apixaban and Warfarin was compared across 3 risk categories: low risk (0), moderate risk (1–2), and high risk (≥ 3). In the Apixaban group (N = 100), the majority of patients were classified as high risk (60.0%), followed by moderate risk (37.0%) and low risk (3.0%). In contrast, the Warfarin group (N = 43) had a greater proportion of patients in the high-risk category (67.5%), with moderate risk at 30.2% and low risk at 2.3%. Regarding thrombotic events, no events were reported in the low-risk and high-risk groups for Apixaban, and only 2 patients in the moderate-risk group (5.4%) had thrombotic events. For Warfarin, no thrombotic events occurred in the low-risk group, while 1 patient (7.7%) in the moderate-risk group and 2 patients (6.9%) in the high-risk group experienced thrombotic events. The *P* values for the comparison of thrombotic events across the CHA_2_DS_2_-VASc risk categories were .176 for Apixaban and .958 for Warfarin, indicating no significant differences in thrombotic event rates between the risk groups for either medication.

### 3.3. Secondary endpoint

#### 3.3.1. The comparison of major and minor bleeding events within 90 days of starting anticoagulation therapy with apixaban versus warfarin showed no significant differences between the 2 groups (Figs. 4 and 5)

**Figure 4.**
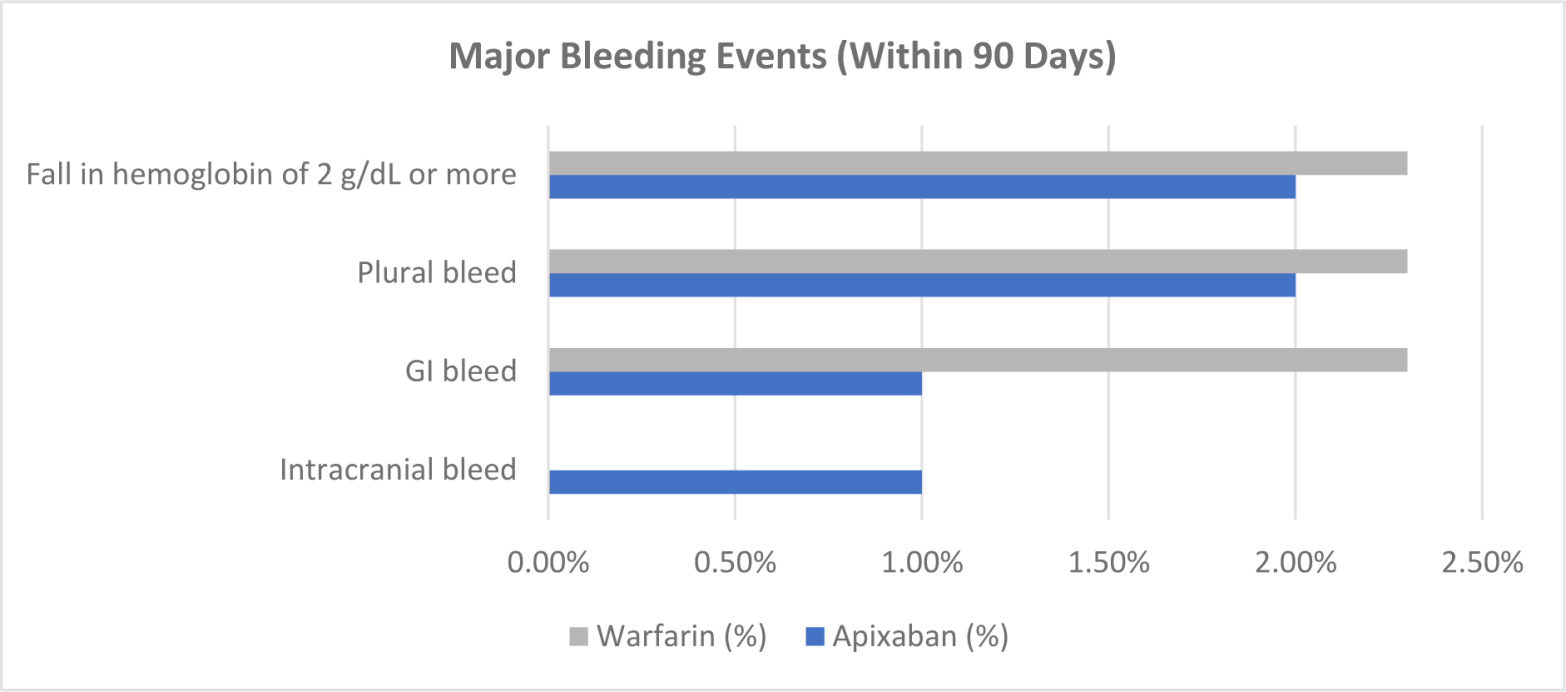
Major bleeding events within 90 days of anticoagulant initiation. GI = gastrointestinal.

For major bleeding (Fig. 4), 6.0% of apixaban patients experienced events, compared to 7.0% of warfarin patients (*P* = .825). The types of major bleeds included intracranial hemorrhage, gastrointestinal bleeding, pleural bleeding, and a significant drop in hemoglobin of 2 g/dL or more.

**Figure 5.**
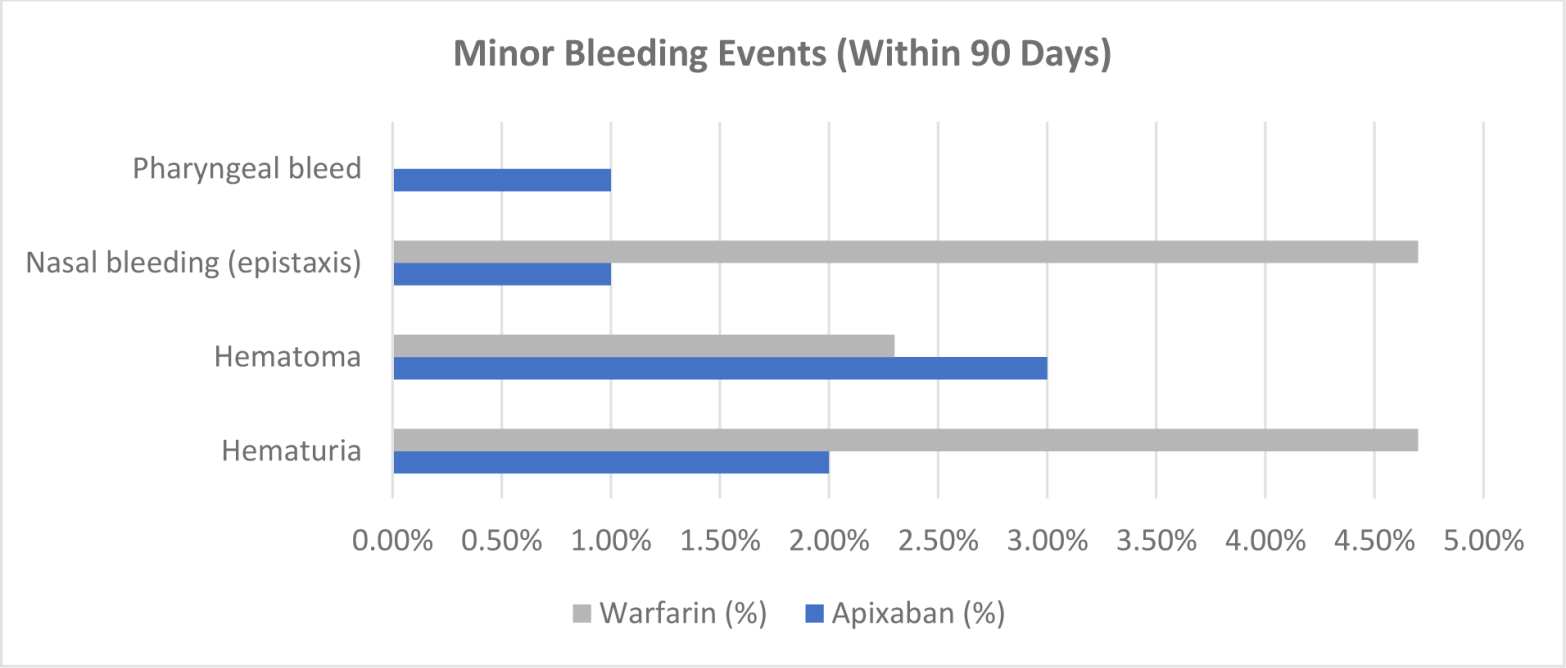
Minor bleeding events within 90 days of anticoagulant initiation.

The rates of minor bleeding (Fig. 5) were 7.0% for apixaban and 11.6% for warfarin (*P* = .360), with minor events such as hematuria, hematoma, nasal bleeding (epistaxis), and pharyngeal bleeding. Although warfarin showed a slightly higher rate of minor bleeding, this difference was not statistically significant.

#### 3.3.2. Post-discharge outcomes

The post-discharge outcomes compare various post-discharge outcomes within 90 days for patients receiving apixaban (N = 100) versus warfarin (N = 43). The outcomes assessed include pericardial effusion, pleural effusion, recurrent AF, rehospitalization rates, and mortality. (Table 6) compares the post-discharge outcomes within 90 days for patients treated with apixaban and warfarin. Regarding pericardial effusion, apixaban showed a higher incidence (10.0%) compared to warfarin (2.3%), although this difference was not statistically significant (*P* = .286). The incidence of pleural effusion was marginally greater in the warfarin group (7.0%) compared to apixaban (6.0%); however, no statistical comparison was provided.

**Table 6:** Post-discharge outcomes for apixaban vs warfarin.

Variables	Apixaban (N = 100)	Warfarin (N = 43)	*P* value
	n (%)	n (%)	
Pericardial effusion after discharge within 90 d	10 (10.0)	1 (2.3)	.286
Pleural effusion after discharge within 90 d	6 (6.0)	3 (7.0)	.217
Recurrent AF after 90 days or the first appointment	7 (7.0)	3 (7.0)	.996
Any rehospitalization after discharge within 90 d	48 (48.0)	14 (32.6)	.088
Rehospitalization for bleeding or thrombotic events	12 (12.0)	2 (4.7)	.175
Death after discharge within 90 d	3 (3.0)	1 (2.3)	.823
Unstable INR or TTR < 60%		34 (79.1)	

AF = atrial fibrillation, INR = international normalized ratio, N/n = number of participants, TTR = time in therapeutic range.

In terms of recurrent AF, both groups had the same rate (7.0%), and the difference was not significant (*P* = .996). A greater proportion of apixaban patients were rehospitalized within 90 days (48.0%) compared to warfarin (32.6%), though this difference did not reach statistical significance (*P* = .088). Apixaban patients also had a higher rate of rehospitalization for bleeding or thrombotic events (12.0%) compared to warfarin (4.7%); however, the difference was not statistically significant (*P* = .175).

Regarding mortality, 3.0% of patients in the apixaban group and 2.3% in the warfarin group died within 90 days, with no significant difference between the groups (*P* = .823).

Lastly, a major issue with warfarin therapy was observed in the form of unstable international normalized ratio or time in therapeutic range (TTR) < 60%, which affected 79.1% of patients on warfarin, emphasizing the difficulty in maintaining therapeutic anticoagulation control in this group.

#### 3.3.2. Predictors for rehospitalization within 90 days for all patients

In the adjusted logistic regression model, chronic kidney disease (CKD) was independently associated with rehospitalization within 90 days after discharge. Patients with CKD had higher odds of rehospitalization compared with those without CKD (adjusted odds ratio 3.90, 95% confidence interval 1.30–11.66; *P* = .015). No other variables were significantly associated with rehospitalization in the adjusted analysis. (See Table S1, Supplemental Digital Content 1).

#### 3.3.3. Predictors for pericardial effusion within 90 days for all patients:

CKD was also independently associated with pericardial effusion within 90 days after discharge. Patients with CKD had significantly higher odds of developing pericardial effusion compared with patients without CKD (adjusted odds ratio 7.89, 95% confidence interval 1.95–31.90; *P* = .004), after adjustment for demographic, clinical, and surgical factors. (See Table S2, Supplemental Digital Content 2).

## 4. Discussion

This study aimed to evaluate the efficacy and safety of apixaban compared to warfarin in patients with new-onset POAF following CABG. It is a well-recognized complication of cardiac surgery, affecting a significant proportion of patients and leading to increased morbidity and healthcare costs (Bachar & Manna, 2023).^[1]^ Our results are consistent with prior research that highlights the challenges of managing AF in this postoperative setting (McIntyre, 2023).^[2]^

In our study, the incidence of thromboembolic events was slightly lower in the apixaban group compared to the warfarin group, though this difference was not statistically significant. These results align with a meta-analysis by Koh et al 2022,^[9]^ which found that DOACs, including apixaban, were associated with lower rates of neurological events and bleeding compared to warfarin. The study reported relative risks of 0.63 for neurological events and 0.74 for major bleeding, suggesting the potential of DOACs to provide a more favorable safety profile in this patient population.^[9]^

In terms of safety outcomes, our study observed comparable rates of major and minor bleeding events between the 2 groups, consistent with findings from Naik et al 2022,^[12]^ who noted similar safety profiles for apixaban and warfarin in patients undergoing cardiac surgery. The incidence of major bleeding was numerically lower in the apixaban group compared with the warfarin group, although the difference was not statistically significant. This supports the notion that DOACs may offer a safer alternative to traditional anticoagulants, particularly in populations at risk for bleeding complications (Sezai et al, 2021; Naik et al, 2022).^[10,12]^

Interestingly, while the CHA_2_DS_2_-VASc scores indicated a high-risk population in both groups, the incidence of thrombotic events was notably low across all risk categories, suggesting that both anticoagulation strategies were effective in this postoperative setting. This is consistent with findings from (Manuel et al 2020),^[11]^ which highlighted that both apixaban and warfarin could be viable options for managing POAF.

In our cohort, 79.1% of warfarin-treated patients had a TTR below 60%. This finding is in line with Manuel et al, who reported delayed achievement of therapeutic anticoagulation with warfarin after cardiac surgery,^[11]^ and underscores a practical limitation of warfarin in the early postoperative period. Pereira et al similarly reported that DOACs were more cost-effective than warfarin in POAF after CABG.^[13]^

The association between CKD and early rehospitalization likely reflects the increased clinical complexity of this patient population. Patients with CKD often have multiple comorbid conditions, require frequent medication adjustments, and are vulnerable to fluid, electrolyte, and metabolic disturbances, all of which may contribute to clinical instability after discharge and increase the likelihood of readmission (Kushwaha et al, 2023; Schulman et al, 2023).^[16,17]^ Similarly, the observed relationship between CKD and pericardial effusion is clinically plausible, as impaired fluid regulation and heightened inflammatory or cardiovascular risk in CKD may predispose patients to effusion development in the post-discharge period (Imazio & Adler, 2013; Gunukula & Spodick, 2001).^[18,19]^ Together, these findings suggest that CKD identifies a high-risk subgroup with increased susceptibility to multiple post-discharge complications. Recognizing CKD during discharge planning may therefore be critical for risk stratification and for guiding targeted interventions, such as closer follow-up, enhanced medication management, and proactive monitoring of fluid status, to reduce adverse outcomes after discharge.

The guidelines from the American College of Cardiology and American Heart Association recommend the use of anticoagulation in patients with POAF to prevent thromboembolic events, highlighting that the choice of anticoagulant should consider individual patient characteristics, including renal function and bleeding risk (Joglar et al, 2024).^[6]^ Our findings reinforce this guidance, suggesting that both apixaban and warfarin can be effective options, though apixaban may provide advantages in terms of convenience and safety.

Furthermore, our findings on rehospitalization rates emphasize the need for further research into the long-term outcomes of these patients. Increased rehospitalizations may reflect complications related to anticoagulation management or inherent surgical risks. Although our study adds to the growing body of evidence supporting the use of apixaban in the postoperative setting, the findings are limited by the small sample size and retrospective design. Moreover, more robust studies are needed to confirm these results.

CKD was identified as a consistent and independent predictor of adverse post-discharge outcomes in our study. In the adjusted analysis, CKD was significantly associated with both rehospitalization and pericardial effusion within 90 days after discharge. In contrast, no other demographic, clinical, or surgical factor showed an independent association with these outcomes.

## 5. Limitations

Despite the insights gained from our study, there are several limitations that should be considered when interpreting its findings. First, the retrospective observational design of the study inherently introduces the potential for biases and confounding factors that are difficult to control. A prospective, randomized controlled trial would provide stronger evidence to compare the efficacy and safety of apixaban and warfarin in this patient population.

Second, this was a single-center study conducted at a tertiary healthcare hospital, which may limit the generalizability of the findings to other settings and populations.

Third, the relatively small sample size, with 100 patients in the apixaban group and 43 in the warfarin group, may have limited the statistical power to detect significant differences between the 2 groups. Larger studies are needed to confirm the study findings. Furthermore, the study did not assess patient adherence to the prescribed anticoagulation regimens, which can impact the observed outcomes. Lastly, the difference in the complexity of the surgical procedures between the 2 groups is an important confounding factor that may have influenced the observed outcomes. Patients undergoing combined CABG and valve surgery tend to have a higher risk profile and potentially a more complicated outcome. Despite adjusting for relevant clinical characteristics, there may be other unmeasured confounding factors that could have influenced the study outcomes.

## 6. Conclusion

This study showed that apixaban and warfarin have comparable effectiveness in preventing thromboembolic events and similar safety profiles concerning bleeding risks for patients with POAF after CABG. The high proportion of warfarin-treated patients with subtherapeutic TTR further supports apixaban as a practical alternative in this setting. Future research should prioritize larger sample sizes and multicenter trials to determine the optimal anticoagulation strategy for patients who develop postoperative AF following cardiac surgery.

## Author contributions

**Conceptualization:** Majed Almutairi.

**Data curation:** Omar S. Alkhezi, Bijesh Kumar.

**Formal analysis:** Omar S. Alkhezi, Bijesh Kumar, Marwan A. Alrasheed.

**Investigation:** Omar S. Alkhezi, Mansour Alomran, Bijesh Kumar, Marwan A. Alrasheed.

**Methodology:** Mansour Alomran, Rawan A. Bukhari.

**Project administration:** Majed Almutairi.

**Software:** Omar S. Alkhezi, Bijesh Kumar.

**Supervision:** Majed Almutairi, Hind Almodaimegh.

**Validation:** Omar S. Alkhezi, Mansour Alomran, Hind Almodaimegh.

**Visualization:** Majed Almutairi, Sultan Alraddadi, Mansour Alomran, Hind Almodaimegh.

**Writing – original draft:** Rawan A. Bukhari, Majed Almutairi.

**Writing – review & editing:** Rawan A. Bukhari, Majed Almutairi, Sultan Alraddadi, Omar S. Alkhezi, Marwan A. Alrasheed.





## References

[1] Bachar BJ Manna B. Coronary Artery Bypass Graft. In: StatPearls - NCBI Bookshelf. StatPearls Publishing; 2023. https://www.ncbi.nlm.nih.gov/books/NBK507836/. 29939613

[2] McIntyre WF. Post-operative atrial fibrillation after cardiac surgery: challenges throughout the patient journey. Front Cardiovasc Med. 2023;10:1156626. 36960472 10.3389/fcvm.2023.1156626 PMC10027741

[3] Burrage PS Low YH Campbell NG O’Brien B. New-onset atrial fibrillation in adult patients after cardiac surgery. Curr Anesthesiol Rep. 2019;9:174–93. 31700500 10.1007/s40140-019-00321-4 PMC6837869

[4] Bessissow A Khan J Devereaux PJ Alvarez‐Garcia J Alonso‐Coello P. Postoperative atrial fibrillation in non‐cardiac and cardiac surgery: an overview. J Thromb Haemost. 2015;13:S304–12. 26149040 10.1111/jth.12974

[5] Oraii A Masoudkabir F Pashang M. Effect of postoperative atrial fibrillation on early and mid-term outcomes of coronary artery bypass graft surgery. Eur J Cardiothorac Surg. 2022;62:ezac264. 35441680 10.1093/ejcts/ezac264

[6] Joglar JA Chung MK Armbruster AL. 2023 ACC/AHA/ACCP/HRS guideline for the diagnosis and management of atrial fibrillation. J Am Coll Cardiol. 2024;83:109–279. 38043043 10.1016/j.jacc.2023.08.017 PMC11104284

[7] Patel S Singh R Preuss CV Patel N. Warfarin. In: StatPearls - NCBI Bookshelf. StatPearls Publishing; 2024. https://www.ncbi.nlm.nih.gov/books/NBK470313/. 29261922

[8] Agrawal A Manna B. Apixaban. In: StatPearls - NCBI Bookshelf. StatPearls Publishing; 2024. https://www.ncbi.nlm.nih.gov/books/NBK507910/. 29939687

[9] Koh KK Ling RR Tan SYS. Direct oral anticoagulants in atrial fibrillation following cardiac surgery: a systematic review and meta-analysis with trial sequential analysis. Br J Anaesth. 2022;129:154–62. 35729010 10.1016/j.bja.2022.05.010

[10] Sezai A Taoka M Osaka S. A comparative prospective observational study on the use of direct oral anticoagulants after cardiac surgery for the management of atrial fibrillation. Ann Thorac Cardiovasc Surg. 2021;27:191–9. 33208579 10.5761/atcs.oa.20-00213 PMC8343030

[11] Manuel L Fong LS Ang ZH Grant P. Comparison of novel oral anticoagulants versus warfarin for post-operative atrial fibrillation after coronary artery bypass grafting. Ann Med Surg (Lond). 2020;58:130–3. 32983432 10.1016/j.amsu.2020.09.007 PMC7493037

[12] Naik KD Whitson BA McLaughlin EM Matre NB Rozycki AJ. Safety of apixaban and rivaroxaban compared to warfarin after cardiac surgery. J Card Surg. 2022;37:4740–7. 36478440 10.1111/jocs.17203 PMC10107629

[13] Pereira MP Lima EG Pitta FG. Rivaroxaban versus warfarin in postoperative atrial fibrillation: cost-effectiveness analysis in a single-center, randomized, and prospective trial. JTCVS Open. 2023;15:199–210. 37808050 10.1016/j.xjon.2023.05.006 PMC10556832

[14] Vahdatpour C Collins D Goldberg S. Cardiogenic shock. J Am Heart Assoc. 2019;8:e011991. 30947630 10.1161/JAHA.119.011991 PMC6507212

[15] Franco L Becattini C Beyer‐Westendorf J. Definition of major bleeding: prognostic classification. J Thromb Haemost. 2020;18:2852–60. 32767653 10.1111/jth.15048

[16] Kushwaha R Vardhan PS Kushwaha PP. Chronic kidney disease interplay with comorbidities and carbohydrate metabolism: a review. Life (Basel). 2023;14:13. 38276262 10.3390/life14010013 PMC10817500

[17] Schulman IH Chan K Der JS. Readmission and mortality after hospitalization with acute kidney injury. Am J Kidney Dis. 2023;82:63–74.e1. 37115159 10.1053/j.ajkd.2022.12.008 PMC10293057

[18] Imazio M Adler Y. Management of pericardial effusion. Eur Heart J. 2013;34:1186–97. 23125278 10.1093/eurheartj/ehs372

[19] Gunukula SR Spodick DH. Pericardial disease in renal patients. Semin Nephrol. 2001;21:52–6. 11172559 10.1053/snep.2001.18378

