# Supplementary material for: Efficacy and safety of apixaban versus warfarin in new-onset atrial fibrillation post coronary artery bypass grafting: A retrospective cohort study
Source: Medicine (Baltimore). 2026 Jul 10;105(28):e49695. doi: 10.1097/MD.0000000000049695 (PMC13362851; doi:10.1097/MD.0000000000049695)
Supplement: Supplementary file 1 [file medi-105-e49695-s001.docx]

Supplementary Tables

Table S1. Any Rehospitalization After Discharge Within 90 Days

| **Variable** | **Estimate**  (Coefficient) | **Std. Error**  (Standard Error) | **Z value** | **P value** |
| --- | --- | --- | --- | --- |
| **Age** | 0.003 | 0.02 | 0.15 | 0.88 |
| **Gender** | -0.03 | 0.50 | -0.065 | 0.95 |
| **BMI** | -0.05 | 0.04 | -1.15 | 0.25 |
| **Heart failure** | 0.84 | 0.61 | 1.37 | 0.17 |
| **Diabetic mellites** | -0.22 | 0.50 | -0.45 | 0.65 |
| **Hypertension** | 0.06 | 0.49 | 0.13 | 0.90 |
| **History of Stroke** | -0.29 | 0.68 | -0.44 | 0.66 |
| **Chronic kidney disease** | 1.36 | 0.56 | 2.43 | 0.01 |

BMI: Body mass index
