# Supplementary material for: Efficacy and safety of apixaban versus warfarin in new-onset atrial fibrillation post coronary artery bypass grafting: A retrospective cohort study
Source: Medicine (Baltimore). 2026 Jul 10;105(28):e49695. doi: 10.1097/MD.0000000000049695 (PMC13362851; doi:10.1097/MD.0000000000049695)
Supplement: Supplementary file 2 [file medi-105-e49695-s002.docx]

Supplementary Tables

Table S2. Pericardial Effusion After Discharge Within 90 Days

| **Variable** | **Estimate**  (Coefficient) | **Std. Error**  (Standard Error) | **Z value** | **P value** |
| --- | --- | --- | --- | --- |
| **Age** | 0.02 | 0.04 | 0.57 | 0.57 |
| **Gender** | 0.02 | 0.74 | 0.03 | 0.98 |
| **BMI** | -0.08 | 0.07 | -1.08 | 0.28 |
| **Heart failure** | 1.39 | 0.82 | 1.70 | 0.09 |
| **Diabetic mellites** | -0.12 | 0.74 | -0.16 | 0.87 |
| **Hypertension** | 0.73 | 0.91 | 0.81 | 0.42 |
| **History of Stroke** | -1.73 | 1.32 | -1.32 | 0.19 |
| **Chronic kidney disease** | 2.07 | 0.71 | 2.90 | 0.004 |

BMI: Body mass index
